# Two-year longitudinal and prospective electroencephalographic follow-up in patients with TBI: can early EEG and CT findings predict post-traumatic epilepsy?

**DOI:** 10.3389/fneur.2025.1609733

**Published:** 2025-07-25

**Authors:** João Paulo Santiago de Oliveira, Viviam Sanabria, Carla Baise, Rafael P. S. Valeriano, Gustavo Mercenas dos Santos, Natália Mata Longo, Joaquina C. Q. F. Andrade, Gesael Passos Ferreira Junior, Carlos André Oshiro, Claudia Costa Leite, Maira L. Foresti, Luiz Eugênio Mello, Eliana Garzon

**Affiliations:** ^1^Division of Neurologic Clinic, Department of Neurology, Faculdade de Medicina, Hospital das Clínicas, Universidade de São Paulo, São Paulo, Brazil; ^2^Laboratory of Neurobiology, Department of Physiology, Universidade Federal de São Paulo, São Paulo, Brazil; ^3^Radiology Institute, Faculdade de Medicina, Hospital das Clínicas, Universidade de São Paulo, São Paulo, Brazil; ^4^Instituto D'Or de Pesquisa e Ensino, São Paulo, Brazil; ^5^Sociedade Beneficente de Senhoras Hospital Sírio-Libanês, São Paulo, Brazil

**Keywords:** traumatic brain injury, post-traumatic epilepsy, seizure, electroencephalography, EEG, biomarker

## Abstract

**Introduction:**

Traumatic brain injury (TBI), caused by external force to the head, leads to anatomical or functional damage to cranial structures. It is a leading cause of morbidity and mortality in adults worldwide, with substantial economic burden. Post-traumatic epilepsy (PTE) is a significant complication of TBI, posing immense challenges to rehabilitation and exacerbating socioeconomic burdens. The incidence of PTE varies widely, underscoring the need for early detection and treatment.

**Objective:**

Through prospective electroencephalography (EEG) evaluations over a two-year period, our study aims to identify electrographic patterns indicative of PTE development, offering crucial insights for timely intervention and improved patient outcomes.

**Methods:**

Seventy-three adult participants with acute TBI, admitted to a reference hospital in Brazil between 2018 and 2020, were recruited based on eligibility criteria. EEG evaluations monitored seizure occurrence with follow-ups for up to 24 months post-TBI, though these were disrupted by the COVID-19 pandemic. Analyses included established EEG protocols, examining factors such as background activity and epileptiform paroxysms. Relative risk (RR), Multiple Correspondence Analysis (MCA), logistic regression, and Generalized Estimating Equations (GEE) were employed to predict variables associated with PTE development.

**Results:**

Both PTE and NO-PTE (no post-traumatic epilepsy) patients showed improving background activity over 2 years. EEG recordings revealed that injuries in the temporal region, diffuse theta waves and abnormal bilateral sleep elements indicated a higher risk of PTE development. Additionally, multiple lesions were also associated with PTE.

**Conclusion:**

This comprehensive approach provides valuable insights for clinical management and sheds light on the complex interplay of factors influencing TBI outcomes.

## Introduction

Traumatic brain injury (TBI) results from external force to the head, causing anatomical or functional impairment of cranial or brain structures. TBI is a leading cause of morbidity and mortality, particularly in adults under 45 (1). It significantly impacts functional capacity and quality of life (2). In the USA, TBI leads to 2.8 million emergency visits and 124,000 severe cases annually (3). In Europe, TBI accounts for 37% of injury-related deaths and costs an estimated 22.9 billion euros annually (4, 5). In Brazil, TBI hospitalizations cost approximately $70.96 million annually (6); however, estimates may not accurately reflect reality due to underreporting of cases and limited epidemiological studies in developing countries.

Epileptic seizures are a major challenge for TBI survivors (7). Post-traumatic epilepsy (PTE) complicates rehabilitation, impacting quality of life and incurring costs for tests, medical visits, medications, and lost workdays, alongside emotional and social impacts (8). After TBI, seizures can be classified as acute symptomatic seizures (ASS), which include both hyperacute post-traumatic seizures (within 24 h of TBI) and early post-traumatic seizures (within 7 days of TBI) (9–11). Seizures occurring beyond 7 days of TBI are classified as late post-traumatic seizures or PTE, indicating ongoing epileptogenesis (12). The incidence of PTE ranges from 0.4 to 26.7%, depending on the study population, TBI severity, and the presence, number, and location of injuries (13, 14). Late post-traumatic seizure (LPTS) or PTE requires early diagnosis and treatment. While no definitive biomarkers exist for predicting PTE, research is ongoing (15). Most cases develop within the first 2 years post-injury. Though an EEG cannot rule out epilepsy, it can identify interictal abnormalities and electrographic seizures (16). Few studies use EEG prospectively after TBI to evaluate epileptogenesis or identify PTE risk factors (15, 17–20). Additionally, longitudinal evaluations of EEG findings in TBI patients are scarce.

The objective of this study was to prospectively evaluate electroencephalographic data from patients who suffered moderate and severe TBI, from the initial acute phase for a two-year follow-up period. We aimed to assess electrographic evolution and identify potential electrographic findings that could predict the occurrence of PTE in these patients.

## Methodology

This study was carried out at the Clinic Hospital of the Faculty of Medicine of the University of São Paulo, Brazil, with approval from the ethics and research committee (CAAEs: 08533513.6.2002.0068 and 08533513.6.1001.5505). Participants were recruited between February 2018 and March 2020.

Inclusion criteria were patients of both sexes, aged between 18 and 75 years, with signed informed consent (by patient or guardian), diagnosed with acute TBI, with acute intracranial hemorrhage and/or contusion confirmed by head computed tomography (CT), and/or with Glasgow Coma Scale (GCS) ≥ 6 and ≤ 12 on admission or GCS < 6 if sedated. Exclusion criteria included a history of epilepsy, use of antiseizure medication, perinatal injuries, meningitis, encephalitis, neoplasia, neurodegenerative diseases, stroke, cognitive dysfunction (dementia), and pregnancy.

Seizure occurrences were classified as hyperacute post-traumatic seizures (within 24 h of TBI), early post-traumatic seizures (within 7 days of TBI), and late post-traumatic seizures (beyond 7 days of TBI), also defined as PTE. Latency for PTE onset, clinical ictal manifestations, and seizure count were noted. Epileptic seizures were classified per International League Against Epilepsy guidelines (21).

Patients underwent an electroencephalogram (EEG) as early as possible during hospitalization, with recordings lasting 1 h each. Clinical conditions and procedures sometimes prevented early recordings in the acute phase. All EEGs were conducted using a 32-channel digital electroencephalograph (Nihon Kohden system, software EEG-1200), with electrodes placed according to the international 10–20 system and fixed with Elefix paste. The recordings used a 1,000 Hz sampling rate, a 0.3 s time constant, and a 70 Hz high-filter. Two experienced board-certified neurophysiologists analyzed the recordings. Cohen’s kappa coefficient was used to evaluate inter-rater agreement, and any discrepancies were resolved by a third board-certified examiner to reach a consensus.

Due to the COVID-19 pandemic starting March 2020, our TBI follow-up protocol, including clinical visits and EEG recordings at specified intervals (1, 3, 6, 12, 18, and 24 months post-injury), was disrupted. New patient recruitment ceased temporarily, and in-person visits and EEGs were suspended. From September 2020 onwards, follow-up activities partially resumed via teleconsultation, with EEG recordings gradually reintroduced under safety protocols. These interruptions caused delays of 1–3 months in data collection, resulting in EEG recordings being classified within pm 3 months of the scheduled intervals. Consequently, some patients were followed for up to 32 months to compensate for overlapping delays caused by the pandemic. For consistency, EEG recording intervals were grouped as follows: acute phase < 7 days (0–7 days); 1 month (7 days–1 month); 3 months (2–4 months); 6 months (5–8 months); 12 months (9–15 months); 18 months (16–22 months) and 24 months (23–32 months).

EEG analysis was performed by visual examination, and the frequencies that constituted the background activity were analyzed and semi-quantified (delta < 4 Hz; theta 4–7.9 Hz; alpha 8–13 Hz; and beta >13 Hz). Delta frequency presence was categorized as absent, rare (<1%), occasional (1–9%), frequent (10–49%), abundant (50–89%), or continuous (>90%). Other parameters included asymmetries, predominant background frequency when awake or after stimulation (beta, alpha, theta, and delta), occurrence of theta or delta in bursts, presence of posterior dominant rhythm and sleep spindle, epileptiform discharges, and electrographic or electroclinical seizures. Electrographic findings adhered to American Clinical Neurophysiology Society guidelines (22).

All patients underwent an initial head CT scan, which served as the primary imaging modality for correlation with other data. Intraparenchymal injuries (contusions and hematomas) were categorized as none, single, dual, or multiple. Lesions were classified by laterality (unilateral or bilateral) and location (frontal, parietal, temporal, occipital). Additional classifications included skull fractures, subdural hematoma (SDH), subarachnoid hemorrhage (SAH), epidural hematoma (EDH), and presence of midline shift (≥ 5 mm). We primarily utilized computed tomography (CT) for evaluations due to the emergency nature of brain trauma care; patients often present with hemodynamic instability or decreased consciousness, necessitating a rapid and readily accessible imaging modality. Thus, the use of magnetic resonance imaging (MRI) for these evaluations was not feasible due given these technical constraints.

### Statistical analysis

Data were tested for normality and homogeneity. Continuous variables were presented as mean ± standard deviation, and categorical variables were described as distribution and frequency. Associations with PTE presence or absence were analyzed using Risk Ratio (RR) and Fisher’s exact test, with a 95% confidence interval (CI).

To estimate the risk of developing PTE from the first month post-injury, we analyzed EEG recordings and CT features obtained within this initial period. Given the predominance of categorical variables and limited sample size, Multiple Correspondence Analysis (MCA) was applied to explore associations among predictors and reduce dimensionality. Based on the MCA results, variables contributing minimally were excluded from further modeling. The retained MCA dimensions were then used as input in a logistic regression model. Included predictors were age, GCS score, number and lateralization of lesions, and EEG findings, classified as normal or abnormal, along with assessments of background activity and interhemispheric symmetry.

Afterward, to investigate the correlation of repeated EEG measurements over time, we performed another MCA with all EEG data (background activity, predominant frequency, sleep spindles, etc.) and variables contributing minimally were excluded from further modeling. Then, we employed a Generalized Estimating Equation (GEE) model with a Poisson distribution, suitable for modeling count or rate-based data. We opted for a Poisson GEE model with a log link and robust standard errors due to the small number of PTE cases (*n* = 9) compared to non-PTE cases (*n* = 57), which led to convergence issues when attempting a logistic GEE model. This quasi-likelihood approach allowed us to analyze longitudinal data robustly without strict distributional assumptions for the dependent variable. Our model used PTE as the dependent variable, with the following predictors:, age, EEG results, background activity, predominant frequency observed during wakefulness and post-stimulus, hemisphere, brain region, presence of theta and delta waves, posterior dominant rhythm, sleep spindles, time window of EEG, and seizure occurrence7 beyond 7 days.

All statistical analyses were performed using Prism software (version 8.0.2; GraphPad, United States) and JMP (version 17. SAS Institute Inc., Cary, NC). Statistical significance was set at *p* < 0.05.

## Results

### Demographic data, mechanism, and severity of TBI

During the recruitment period, 123 patients were included. Of this total, 30 (24.4%) patients were excluded for different reasons, such as not meeting the eligibility criteria (at a subsequent evaluation), being transferred to other services, withdrawing informed consent during the follow-up phase, being unable to perform any of the on-site EEG examinations, or being lost during the follow-up period. Twenty-seven (21.9%) patients died, and of those, 20 had no electroencephalographic recording and were, therefore, excluded.

**Figure 1:**
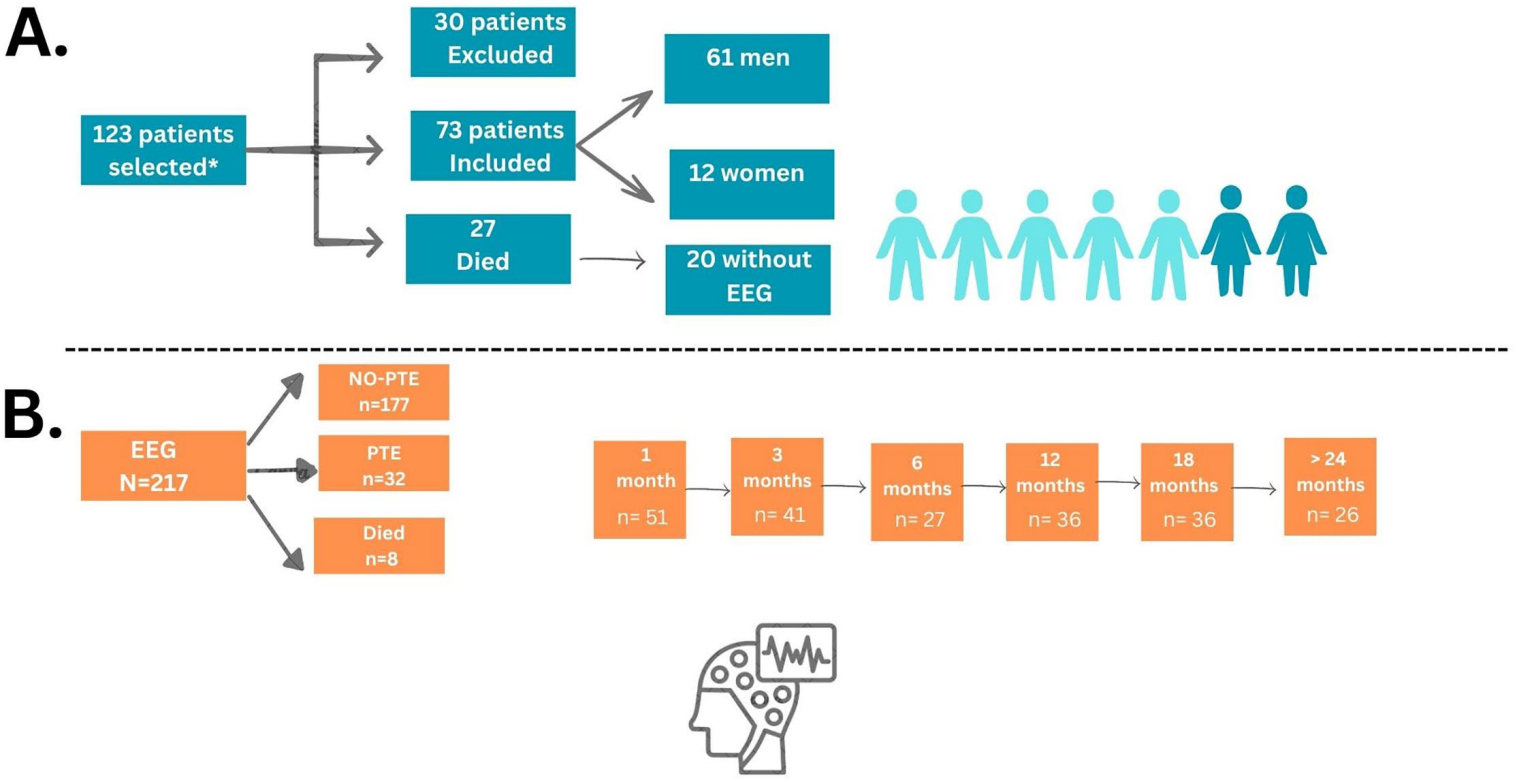
Flowchart of selected patients and EEG. **(A)** *Inclusion criteria comprised patients of both sexes, aged between 18 and 75 years, who provided signed informed consent (either by the patient or a legal guardian), and had a diagnosis of acute TBI confirmed by head CT showing intracranial hemorrhage and/or cerebral contusion. Eligible patients had a GCS score between 6 and 12 upon admission, or a GCS < 6 if sedation was present at the time of evaluation. **(B)** EEG summary follow-ups.

Data from 73 (59.3%) patients (12 women and 61 men) were analyzed (Table 1). The mean age was 43.7 years (range: 18–80 years). The most common cause of TBI was traffic accident (44%), followed by falls (39%), aggression (6%), bicycle crash (6%), falling objects to the head (3%), and unknown mechanism (2%). Most TBIs were moderate and severe (62%), and 26 patients could be followed up for at least 24 months. Among these, 13 patients underwent EEG recordings during the first week, 38 patients at 1 month, 41 patients at 3 months, 27 patients at 6 months, 36 patients at 12 months, 36 patients at 18 months, and 26 patients beyond 24 months (Table 1 and Figure 1).

**Table 1:** Demographics data, TBI mechanisms and severity, ASS in PTE, No-PTE groups and in patients who evolved to death.

Variables	Total participants (*n* = 73)	Death (*n* = 7)	PTE (*n* = 9)	NO-PTE (*n* = 57)
Sex				
Male, n (%)	61 (84)	7 (100)	7 (78)	47 (82)
Female, n (%)	12 (16)	0 (0)	2 (22)	10 (18)
Age (mean ± SD)	44 ± 17.6	47 ± 20.18	37 ± 13.8	40 ± 15.53
TBI Mechanism, n (%)				
Traffic accident	34 (44)	3 (43)	3 (33)	28 (49)
Height falling	21 (29)	1 (14)	5 (56)	15 (26)
Own height falling	7 (10)	1 (14)	0 (0)	6 (11)
Aggression	4 (6)	0 (0)	0 (0)	4 (7)
Falling objects	2 (3)	1 (14)	1 (11)	0 (0)
Bicycle crash	4 (6)	1 (14)	0 (0)	3 (5)
Unknown mechanism	1 (2)	0 (0)	0 (0)	1 (2)
TBI Severity (GSC), n (%)				
Mild	28 (38)	4 (57)	2 (22)	22 (38)
Moderate	6 (8)	1 (14)	0 (0)	5 (9)
Severe	39 (54)	2 (29)	7 (78)	30 (53)
Acute symptomatic seizures (ASS), Yes n (%)				
(0–7 days)	16 (22)	2 (29)	3 (33)	11 (19)
Immediate ASS				
(<24 h)	13 (18)	2 (29)	3 (33)	8 (14)
Early ASS				
(>24 h–7 days)	3 (4)	0 (0)	0 (0)	3 (5)
PTE, Yes				
Time to first PTE seizure (months)				
Average (min-max)	–	**–**	10.9 (1–20.9)*	**–**
Time to first PTE seizure				
< 12 months, n (%)		**–**	3 (33)	**–**
>12–24 months, n (%)		**–**	5 (55)	**–**
≥24 months, n (%)		**–**	1 (11)	**–**
**Number of EEGs at scheduled timepoints	217	8	32	177
Acute phase (<7 days)	13 (5.9)	1 (7.7)	5(38.5)	7 (53.8)
>7 days until 1st month	38 (17.5)	5 (13.2)	3 (7.9)	30 (78.9)
3rd month	41 (18.9)	2 (4.9)	8 (19.5)	31 (75.6)
6st month	27 (12.4)	0 (0)	3 (11.1)	24 (88.9)
12nd month	36 (16.6)	0 (0)	4 (11.1)	32 (88.9)
18th month	36 (16.6)	0 (0)	5 (13.9)	31 (86.1)
≥ 24th month	26 (12.9)	0 (0)	4 (15.4)	22 (84.6)
Seizure frequency				
1 remote symptomatic seizure	–	–	3	–
1–2 seizure/month	–	–	3	–
3–4 seizure/month	–	–	2	–
≥ 5 seizure/month	–	–	1	–

PTE occurrence and time for first seizure. Data for the total and subdivided population according to treatment with No-PTE or PTE for demographics, TBI mechanisms, and severity in patients. Continuous variables were described as mean ± standard deviation (SD), while categorical variables were described as distribution and frequency. *One patient presented a seizure after 25 months of follow-up. **Number of EEG exams performed in each period of time. Note that some patients underwent EEG exams in more than one time period, which is why the number of EEG exams differs from the number of patients.

The 7 patients who died but had at least one EEG at some point of follow-up were analyzed as a separate group. All 7 patients were male, with a mean age of 47.4 ± 20.18 years, with traffic accidents as the mechanism of TBI in 3 patients. Four (57%) of the patients who died had only mild TBI. In general, death occurred 4 months (median) after TBI, ranging from 6 to 383 days of follow-up (one patient who died after 383 days was in supportive care and could not return for follow-up EEGs but was clinically evaluated). Among these patients, 1 patient underwent EEG recordings during the first week, 5 patients at 1 month, and 2 patients at 3 months (Table 1). Despite the small sample size and the limitation of follow-up due to death, which precludes long-term assessment—including the potential development of post-traumatic epilepsy—this group was included to explore whether any electrographic markers could serve as predictors of unfavorable outcomes, such as mortality.

### Acute symptomatic seizure and late onset seizures or PTE

Sixteen patients (22%) had acute symptomatic seizures (ASS): 13 (18%) had hyperacute post-traumatic seizures, and 3 (4%) had early post-traumatic seizures.

Fifty-seven (78%) patients did not develop PTE (NO-PTE group), while nine (12%) patients developed spontaneous seizures after TBI (late-onset post-traumatic seizures, herein after referred to as PTE) (Table 1).

Among patients in the NO-PTE group, 11 (19%) had ASS, 8 of them in the first 24 h after TBI (Table 1). For the PTE group, 3 (33%) also had ASS, all of them occurring within the first 24 h of TBI (Table 1). Similarly, two (28.5%) patients who died also presented immediate ASS (Table 1).

Related to PTE development, the earliest latency for the first spontaneous seizure was 2 months after the TBI, and the latest was 25 months after the TBI. On average, epilepsy started at 10.9 months after TBI (Table 1). Regarding the number of seizures for those who developed PTE, 3 patients (33%) had only 1 seizure, while the other 6 patients reported more than 2 seizures during the follow-up period. Eight patients were using anti-seizure medication by decision of the neurosurgery service; 5 were in the non-PTE group and 3 in the PTE group. No patients who died had developed PTE prior to their death.

### Electroencephalographic recording

A total of 217 electroencephalographic recordings were analyzed for 73 patients: 32 recordings from the PTE group, 177 from the NO-PTE group, and 8 recordings from patients who died during the 2-year follow-up (Table 2). Inter-rater reliability demonstrated almost perfect agreement (*κ* = 0.82, *p* < 0.001).

**Table 2:** EEG findings in PTE, No-PTE groups and in patients who evolved to death during 2 years follow-up.

EEG Findings (number of EEG exams)	Summary of findings (*n* = 217)	Death (*n* = 8)	PTE group (*n* = 32)	NO-PTE group (*n* = 177)
Abnormal EEG	140/217 (64.5)	8/8 (100)	21/32 (65.6)	111/177 (62.7)
1st month	47	6	7	34
3rd month	28	2	7	19
6st month	16	0	1	15
12nd month	20	0	1	19
18th month	16	0	3	13
≥ 24th month	13	0	2	11
Abnormal EEG with delta	27/217 (12.4)	4/8 (50)	7/32 (21.8)	15/177 (8.5)
1st month	20	3	5	12
3rd month	4	1	1	2
6st month	0	0	0	0
12nd month	0	0	0	0
18th month	0	0	0	0
≥ 24th month	3	0	2	1
Abnormal EEG with delta + theta	24/217 (11)	1/8 (12.5)	9/32 (28.1)	14/177 (7.9)
1st month	7	1	2	4
3rd month	8	0	4	4
6st month	2	0	1	1
12nd month	2	0	0	2
18th month	4	0	2	2
≥ 24th month	1	0	0	1
Abnormal EEG with theta	42/217 (19.3)	2/8 (25)	3/32 (6.2)	37/177 (20.9)
1st month	8	1	0	7
3rd month	9	1	2	6
6st month	8	0	0	8
12nd month	8	0	0	8
18th month	6	0	1	5
≥ 24th month	3	0	0	3
Burst-suppression pattern	1/217 (0.46)	1/8 (12.5)	0/32 (0)	0/177 (0)
1st month	1	1	0	0
3rd month	0	0	0	0
6st month	0	0	0	0
12nd month	0	0	0	0
18th month	0	0	0	0
≥ 24th month	0	0	0	0
Presence of the posterior dominant rhythm (≥ 8 Hz)	175/217 (80.6)	1/8 (12.5)	24/32 (75)	150/177 (84.7)
1st month	25	1	2	22
3rd month	33	0	7	26
6st month	27	0	3	24
12nd month	34	0	4	30
18th month	33	0	4	29
≥ 24th month	23	0	4	19
Asymmetry of background activity	86/217 (39.6)	4/8 (50)	17/32 (53.1)	65/177 (36.7)
1st month	30	3	7	20
3rd month	14	1	4	9
6st month	10	0	1	9
12nd month	13	0	2	11
18th month	10	0	1	9
≥ 24th month	9	0	2	7
Bursts of slow waves	42/217 (19.3)	1/8 (12.5)	1/32 (3.1)	40/177 (22.6)
1st month	12	1	0	11
3rd month	7	0	0	7
6st month	5	0	0	5
12nd month	7	0	1	6
18th month	5	0	0	5
≥ 24th month	6	0	0	6
Epileptiform discharges	3/217 (1.3)	0/8 (0)	3/32 (9.3)	0/177(0)
1st month	0	0	0	0
3rd month	1	0	1	0
6st month	0	0	0	0
12nd month	0	0	0	0
18th month	1	0	1	0
≥ 24th month	1	0	1	0

Abnormalities in background activity were observed in 140 of all 217 (64.5%) EEG recordings: 21/32 (65.6%) in the PTE group, 111/177 (62.7%) in the NO-PTE group, and 8/8 (100%) for patients who died. Asymmetric background activity was the most common abnormality, occurring in 40% of all recordings, independent of the group (Table 2). The presence of slow waves (delta, delta mixed with theta) constituting the background activity was higher in the PTE group (Table 2).

Epileptiform abnormalities were found in only 3 EEG recordings from 2 patients in the PTE group.

**Figure 2:**
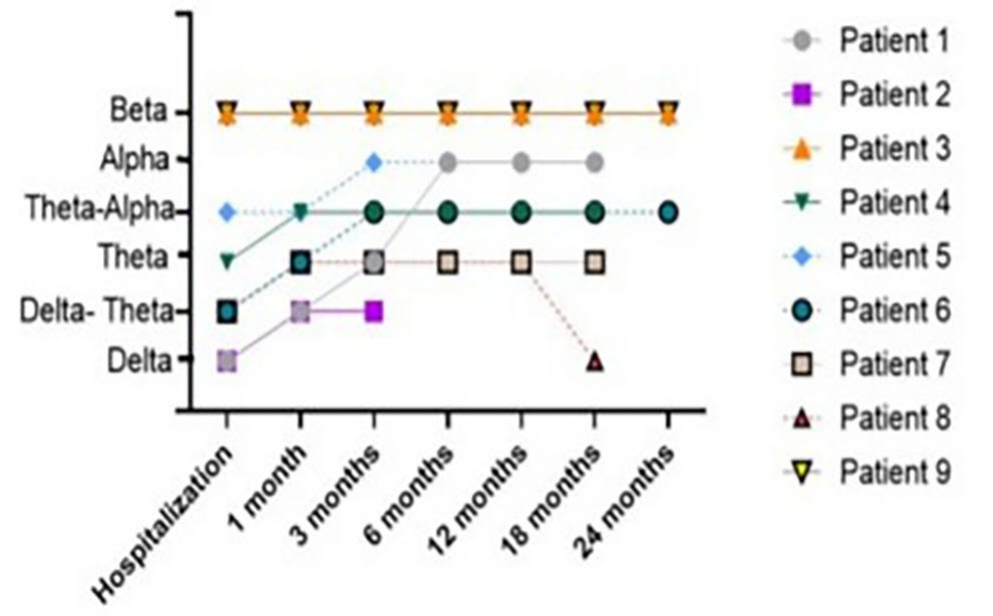
Predominant frequency observed during awake or stimulated and its evolution over 24 months of follow-up in PTE group.

To determine electrographic evolution, the predominant frequency observed during awake or stimulated states was evaluated in all records over 2 years. Up to the sixth month of evolution, in both groups, there was still a considerable number of recordings that exhibited a large number of slow waves. Over time, the number of slow waves dropped progressively, the dominant posterior physiological rhythm became more common, and EEG findings tended to improve over time. Figure 2 illustrates the predominant frequency observed during awake or stimulated states and its evolution over time in the PTE group. All patients showed improvement of background activity, except patient 8, who at 18 follow-up months showed an increase in the number of slow waves. This finding was very close to the occurrence of the first spontaneous seizure.

The number of EEGs from patients who died was small, precluding statistical analysis for this group. However, 100% of the recordings in this group were abnormal, and the only recording with burst suppression was also in this group (Table 2).

### Relationship between EEG, brain CT, and PTE

To explore the relationship between EEG and CT with the occurrence of PTE, we computed Relative Risks (RRs) (Table 3).

**Table 3:** Comparative analysis of EEG and CT findings, Early ASS, TBI severity, in PTE and No-PTE groups.

Variables	PTE (*n* = 9)	NO-PTE (*n* = 57)	RR	95% CI	Fisher’s exact test *p*-value
Normal EEG	11/32	68/177			
Abnormal EEG	21/32	111/177	0.87	0.44–1.68	0.84
Recordings with delta in background activity					
No	14/32	130/177	0.35	0.18–0.66	0.0016**
Yes	18/32	47/177			
Incidence of delta waves					
Rare <1%	4/32	10/177			
Occasional (1–9%)	5/32	19/177	1.37	0.45–4.00	0.69
Frequent (10–49%)	4/32	12/177	1.14	0.36–3.56	> 0.99
Abundant (50–89%)	5/32	6/177	0.91	0.39–2.23	> 0.99
Sleep spindles					
Normal	9/32	62/177	1.31	0.42–4.31	>0.99
Abnormal	3/32	28/177			
Epileptiform discharges					
No	29/32	177/177	0.14	0.099–0.34	**0** .0033**
Yes	3/32	0/177			
Posterior dominant rhythm (≥ 8 Hz)					
Absence	8/32	27/177	1.66	0.80–3.22	0.20
Presence	24/32	150/177			
Symmetry					
Asymmetric	17/32	65/177	1.75	0.93–3.28	0.11
Symmetric	15/32	112/177			
CT findings					
Number of intraparenchymal lesions, n (%)					
One lesion	4 (44.4)	24 (43)			
Two lesions	2 (22.2)	8 (14)	0.7	0.18–3.11	0.64
Multiple lesions	3 (33.3)	17 (29)	0.95	0.26–3.53	> 0.99
Without lesions	0 (0)	8 (14)	Infinity	0.33- Infinity	0.56
Lateralization of lesions, n (%)					
Unilateral	8 (88.8)	33 (58)			
Bilateral	1 (11.1)	16 (28)	3.31	0.62–19.95	0.25
Without lesions	0 (0)	8 (14)	Infinity	0.54- Infinity	0.32
Location of lesions, n (%)					
Frontal	5 (55.5)	32 (56)			
Temporal	7 (77.7)	22 (38)	0.56	0.20–1.52	0.34
Parietal	1 (11.1)	5 (8)	0.81	0.17–5.03	> 0.99
Occipital	1 (11.1)	2 (3)	0.40	0.11–2.47	0.39
Fracture, n (%)					
Yes	6 (66.6)	18 (32)	3.50	1.04–11.89	0.06
No	3 (33.3)	39 (68)			
Other lesions n (%)					
Subdural hematoma (SDH)	4 (44)	27 (47)	0.90	0.28–2.86	>0.99
Subarachnoid hemorrhage (SAH)	6 (66)	32 (56)	1.47	0.44–5.08	0.72
Epidural hematoma (EDH)	2 (22)	18 (31)	0.71	0.16–2.45	0.71
Midline deviation, n (%)					
Yes	3 (33.3)	11 (19.2)	1.73	0.56–4.28	0.39
No	6 (66.6)	46 (80.7)			
Early acute symptomatic seizures (>24–7 days)					
Yes	0	3	Infinity	0.34- Infinity	>0.999
TBI severity (GSC)					
Mild	2	22			
Moderate	0	5	Infinity	0.14-Infinity	>0.999
Severe	7	30	0.44	0.11–1.66	0.46
Seizure type					
Focal-to-bilateral tonic–clonic seizure	6/9				
With Impaired consciousness seizure (Without observable manifestations)	6/9	–			
Unknown whether with impaired consciousness	3/9	**–**			
With observable manifestations (bilateral tonic–clonic seizure)	2/9				
Without observable manifestations	1/9				

Seizure types in PTE group. **p < 0.005.

Recordings showing delta waves in background activity exhibited an RR of 0.35 (95% CI: 0.18–0.65, *p* < 0.01) for PTE compared to those without delta activity. Similarly, the presence of epileptiform discharges was associated with an RR of 0.14 (95% CI: 0.09–0.39, *p* < 0.001) for developing PTE compared to its absence. No significant associations were observed for sleep spindles, asymmetry, or posterior dominant rhythm (Table 3).

Brain CT findings were categorized based on the presence of lesions (single, two, multiple, or no lesion), location (frontal, temporal, parietal, occipital), and laterality (unilateral or bilateral). Additional features such as fracture, subdural hematoma (SDH), subarachnoid hemorrhage (SAH), and epidural hematoma (EDH) were also analyzed in the PTE and NO-PTE groups, but no statistically significant differences were identified (Table 3).

### Seizure types

In the PTE group, focal onset seizures evolving to bilateral tonic–clonic seizures were the most common type, occurring in 6 out of 9 patients. Three patients experienced seizures with unknown onset, of which two presented motor features and one with non-motor features (Table 3).

### Multiple correspondence analysis

Multiple Correspondence Analysis (MCA) was employed to examine the associations among categorical predictors and reduce dimensionality prior to logistic regression and GEE given the predominance of qualitative data and the small sample size.

The first MCA analysis regarding CT features and first month data included: PTE outcome, ASS, EEG features (overall result, background activity, symmetry), sex, GCS, and CT findings (number and lateralization of lesions, SAH, SDH, EDH, and midline shift). Data were visualized in Euclidean space.

A two-dimensional solution was selected (eigenvalues: 3.09 and 1.78; inertia: 0.34 and 0.26; Cronbach’s Alpha: 0.76 and 0.50). Variables with low contributions (mean < 0.1)—SAH, SDH, EDH, midline shift, skull fracture, ASS, location of lesion (parietal, occipital, and frontal), and sex—were excluded from further analysis. The two dimensions accounted for 41.3% of the total variance (Dimension 1: 23.4%; Dimension 2: 17.9%). The resulting two-dimensional plot revealed distinct clustering patterns.

**Figure 3:**
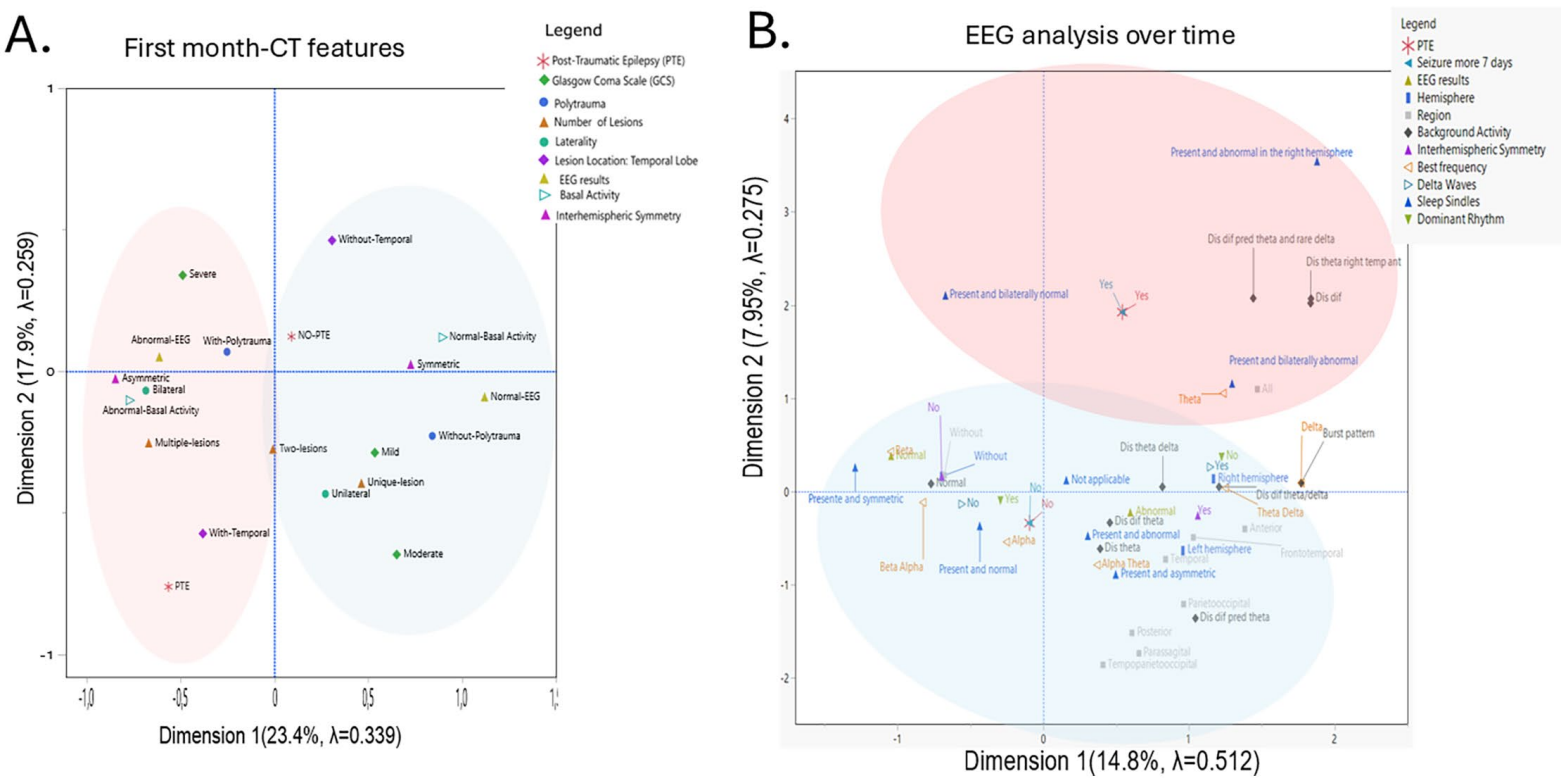
Multiple Correspondence Analysis (MCA) of clinical and neuroimaging. Variables in relation to post-traumatic epilepsy. **(A)** Two-dimensional MCA plot illustrating the associations among categorical clinical, neuroimaging, and Electrophysiological variables in relation to the presence or absence of post-traumatic epilepsy (PTE) during the first month. The first two dimensions explain 41.3% of the total variance (Dimension 1: 23.4%, inertia 0.34; Dimension 2: 17.9%, inertia 0.26). The red circle represents the profile called PTE, and the blue circle represents the profile called No-PTE. **(B)** Two-dimensional MCA plot illustrating the associations among electrophysiological variables about the presence or absence of post-traumatic epilepsy (PTE) over time. The first two dimensions explain 22.75% of the total variance (Dimension 1: 14.8%, inertia 0.51; Dimension 2: 7.95%, inertia 0.27). The red circle represents the profile called PTE, and the blue circle represents the profile called No-PTE. For Background Activity meanings: Dis dif theta: Disorganized diffused theta waves, Dis dif theta/delta: Disorganized diffused theta/delta; Dis theta/delta: Disorganized theta/delta Dis dif pred theta; Disorganized diffused predominant theta. Dis dif: Disorganized and diffused. Dis dif pred theta and rare delta; Disorganized diffused predominant theta and rare delta. Dis theta ant right hemisphere: Disorganized theta in the anterior right temporal.

MCA revealed two profiles: NO-PTE (blue), associated with normal EEG, symmetry, ASS use, mild/moderate GCS, fewer lesions, no polytrauma, and absence of temporal lesions; and PTE (red), linked to bilateral/multiple lesions, presence of temporal lesions, polytrauma, abnormal baseline EEG, and asymmetry (Figure 3A).

The second MCA analysis was performed regarding longitudinal EEG analysis included: PTE outcome, EEG features (overall result, background activity, symmetry), sex, GCS, and predominant or best frequency, delta and theta waves, sleep spindles, epileptiform paroxysm, occurrence of seizures within 24 h, with 7 days and with more than 7 days.

A two-dimensional solution was selected (eigenvalues: 5.63 and 3.02; inertia: 0.51 and 0.27; Cronbach’s Alpha: 0.90 and 0.74). Variables with low contributions (mean < 0.1)—sex, epileptiform paroxysm, seizures within 24 h, seizures occurrence with 7 days, and theta waves —were excluded from further analysis. The two dimensions accounted for 22.75% of the total variance (Dimension 1: 14.8%; Dimension 2: 7.95%). The resulting two-dimensional plot revealed distinct clustering patterns.

MCA revealed two profiles: NO-PTE (blue), associated with present and symmetric sleep spindles, presence of beta, beta-alpha, and alpha frequency with a dominant rhythm; and PTE (red), linked to presence of bilateral normal and abnormal sleep spindles, and disorganized and diffused predominant theta waves, as well as theta and delta frequencies (Figure 3B).

### Logistic regression model

A binomial logistic regression analysis using the backward elimination method was conducted to assess the likelihood of PTE, incorporating various clinical and neuroimaging variables: age, GCS score at hospital admission, presence of ASS, CT features (number of brain lesions, presence of temporal lesions, lesion lateralization), EEG results, background activity, and interhemispheric symmetry in EEG recordings obtained within the first month after TBI.

The overall model demonstrated a good fit, as indicated by a significant likelihood ratio test [χ^2^(8) = 39.77, *p* < 0.0001] and a Cox and Snell R2 of 0.467, suggesting that approximately 46.7% of the variance in PTE outcomes was explained. The Bayesian Information Criterion (BIC) was 37.28, supporting a favorable balance between model fit and complexity. Several variables (EEG results, background activity, ASS, age, and interhemispheric symmetry) were excluded from the final model due to a lack of statistical contribution.

Key predictors retained in the model were GCS, lesion lateralization, presence of temporal lesions, polytrauma, and number of lesions. However, most of these variables did not show statistically significant odds ratios when analyzed individually, likely due to the small number of PTE cases and overlapping effects among predictors.

Significant findings were observed for lesion lateralization (e.g., right vs. left: OR = 10.23, 95% CI: 1.36–120, *p* = 0.043). For right hemisphere lesion vs. bilateral lesions, the Odds Ratio (OR) was 9.15 (95% CI: 1.20–108, *p* = 0.045).

These findings should be interpreted with caution, as the small number of PTE cases and potential overlap among predictors may affect the precision of individual estimates, despite their combined importance in the overall model.

### Generalized estimating equations

We conducted a Generalized Estimating Equations (GEE) analysis with a log link function and a first-order autoregressive [AR(1)] correlation structure to assess factors influencing PTE over 2 years. Measurements were taken at 1, 3, 6, 12, 18, and 24-months post-injury. The model demonstrated a good fit with a quasi-likelihood under the Independence Model Criterion (QIC) score of 9.89. Significant predictors of PTE included background activity (χ^2^ Wald = 205.28, d.f. = 8, *p* < 0.0001), sleep spindles (χ^2^ Wald = 2312.75, d.f. = 7, *p* < 0.0001), and delta waves (χ^2^ Wald = 4.20, d.f. = 1, *p* = 0.04). Multiple comparisons were corrected by Bonferroni test.

Therefore, the model could be represented by the following equation:


$$\begin{array}{l} \mathrm{Log}\;(\mathrm{PTE})=\beta 0+\beta 1(\text{Background Activity})+\beta 2(\text{Sleep Spindles})\\ +\beta 3(\text{Delta waves})+\in \end{array}$$

Where each beta coefficient represents the effect size of the corresponding predictor variable, and epsilon denotes the error term.

Univariate effects were compared by multiple analysis using the Bonferroni test.

**Figure 4:**
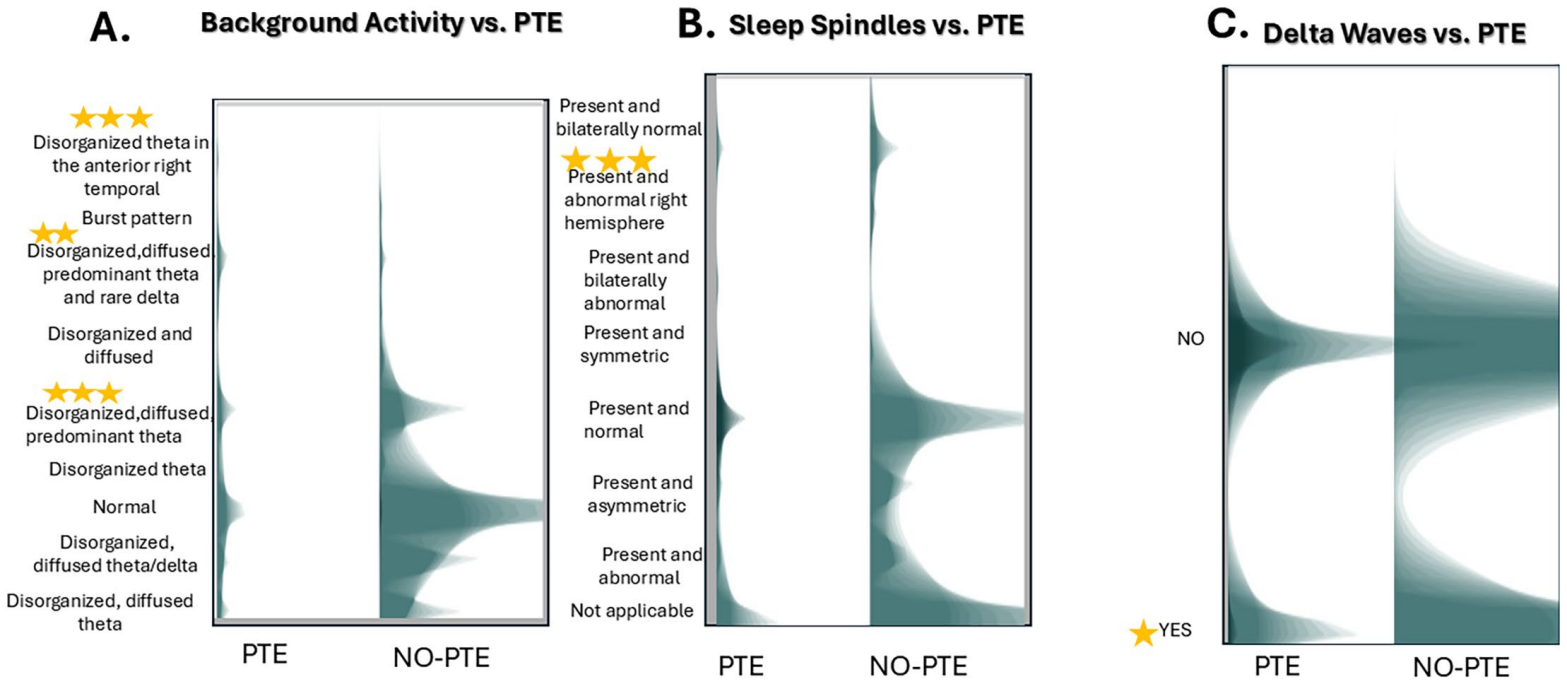
Unimodal associations between EEG features and post-traumatic epilepsy (PTE) risk. Unimodal analysis of EEG predictors for PTE development. **(A)** Background activity. **(B)** Sleep spindle **(C)** Delta wave presence in PTE versus No-PTE cases. Data derived from a GEE model [log link, AR(1) correlation; QIC = 10.18] with Bonferroni-corrected significance. The graph is a histogram of the two variables with a shadowgram style. Stars denote statistical significance. The corrected Significance of every variable is in the text.

- *Background activity:* A one-unit increase in background activity is associated with the following increases in PTE likelihood: 84% for disorganized theta waves in the anterior right hemisphere (*p* = 0.001), 53% for diffuse predominant theta waves with rare delta waves (*p* = 0.006), and 55% for diffuse predominant theta waves (*p* = 0.001) (Figure 4A).
- *Sleep spindles*: A one-unit increase in abnormal sleep elements is associated with an 80% increase in PTE likelihood with the presence of abnormal bilateral sleep elements (*p* < 0.001) (Figure 4B).
- *Delta waves:* A one-unit increase in delta wave presence is associated with a 96% increase in the probability of developing PTE (*p* = 0.020) (Figure 4C).

## Discussion

This study aimed to prospectively evaluate the electroencephalographic data of patients with acute TBI to assess electrographic evolution and identify potential routine EEG findings that could predict PTE. Despite the SARS-CoV-19 pandemic interruptions, we initially recruited 123 patients and prospectively followed 73 of them. Both PTE and NO-PTE patients showed improved background activity over 2 years. EEG recordings revealing delta background indicated a higher risk of PTE development. Additionally, multiple and bilateral lesions are also indicative of a greater probability of developing PTE.

The findings align with existing literature on early seizure incidence and risk factors following severe TBI. The observed ASS incidence (21.9%) falls within the reported range (2–30%) for severe TBI patients (23–26). Notably, a substantial proportion of ASS occurred within the first 24 h post-TBI. Although no statistically significant correlation was found between ASS and PTE development, the higher ASS proportion in the PTE group suggests a potential association, consistent with literature indicating ASS as predictors of later epilepsy development (26, 27). This highlights the complex interplay of injury severity, neuronal circuit disruptions, and genetic predisposition in PTE pathogenesis, beyond the immediate occurrence of ASS.

The PTE incidence in our series was 12.4%, consistent with literature data but lower than expected. PTE represents 20% of all structural causes and 5% of all acquired epilepsies (28). Known risk factors include advanced age, injury severity, intracranial hemorrhage, focal contusion, multiple injuries, coma for over 24 h, loss of consciousness for over 24 h, and prolonged amnesia (7, 13, 29). The PTE incidence varies based on trauma severity and the number of injuries, with cumulative risk ranging from 2 to 50% depending on injury location and severity (7, 24, 25). Following a single late seizure post-TBI, the likelihood of subsequent seizures increases by 80%, typically occurring within 2 years of the first spontaneous late seizure (7). In this study, 22.2% of PTE cases were mild and 77.7% were severe TBI. Among the 9 PTE patients, 6 had more than 2 seizures, with all experiencing only one seizure type.

The mortality rate in TBI is variable, depending on age, trauma severity, and presence of polytrauma, with literature reporting rates from 3.5 to 38.5% (6, 30). In this study, focusing on a small sample of mostly young adults with severe TBI, and mild cases with intraparenchymal injury, the mortality rate was 22.5%. Notably, patients with a GCS ≤ 6 at the scene were not enrolled, potentially excluding those with an even higher likelihood of death.

Electrographic biomarkers may predict seizure onset and epileptogenesis, allowing for the development of targeted preventative therapies. Currently, no validated electrophysiological biomarkers for PTE exist. However, potential markers include pathological high-frequency oscillations (HFOs), reduced sleep spindle duration, changes in theta oscillations, and slower transition from slow wave N3 to REM (31, 32).

Our study aimed to identify electrographic patterns indicative of PTE development through prospective EEG evaluations over 2 years. Initially, many recordings showed delta slow waves, which progressively decreased, reflecting improved EEG frequencies and background activity over time. In the PTE group, one patient showed background activity slowing after 16 months, preceding their first epileptic seizure. Although not statistically significant, these findings suggest that slow waves may indicate dysfunction in epileptogenesis-related circuitry or result from frequent epileptiform discharges or unrecognized seizures. Overall, our EEG data reveal abnormal patterns characterized by background activity replaced by slow waves, loss of physiological sleep patterns, and loss of anteroposterior differentiation while awake.

In clinical practice, epileptiform activities are crucial in evaluating epilepsy or suspected epilepsy, while background rhythms analysis is often undervalued and not correlated with clinical aspects. For instance, a patient with frequent seizures may show slowing or attenuation of physiological rhythms, which can be lateralizing or localizing. Conversely, a patient with controlled seizures may show normal baseline rhythms more frequently. These findings highlight the potential utility of EEG abnormalities as predictors of PTE.

The data found in this study associate epileptiform paroxysms with the risk of progression to PTE. This finding is surprising, given that many patients with refractory epilepsy do not have epileptiform elements during their routine EEG recordings. Often during long-term video-EEG tentative recording of those seizures, several hours or even days may pass without showing any epileptiform elements. The location and depth of the epileptogenic and irritative zone may also not favor surface recordings, and finally, it may be that pathological action potentials are the last phase of the epileptogenic process, with the beginning of epileptic seizures being the completion of the installation of epileptogenesis, which may even continue to evolve after the onset of seizures.

Kim et al. (18) performed a retrospective case–control study of risk factors for post-traumatic epilepsy, in which 25 consecutive cases developed epilepsy within 1 year of a head trauma. They found that EEG epileptiform abnormalities during the acute period post-injury and sporadic epileptiform discharges were associated with PTE.

Regarding the presence of structural injuries, it is already known that the cumulative probability of PTE is higher according to the number of lesions, reaching 25% for multiple lesions (7). In our study, for each brain lesion, individuals are approximately 5.05 times more likely to develop PTE. Individuals with lesions predominantly located on one side of the brain are approximately 0.13 times (or 86% less likely) to develop PTE as compared to those with bilateral lesions.

The data found in this study reinforce the importance of performing routine EEG in the acute phase and in the follow-up of TBI patients. In addition to EEG being the best test for recording epileptic seizures, it is a low-cost test that can be carried out at the bedside without having to transport the patient, who is often unstable, immediately after severe TBI.

Unfortunately, our series was small due to the SARS-CoV-19 pandemic. We acknowledge that the small number of PTE cases (*n* = 9) constitutes a substantial limitation, which may reduce the power to detect true associations and limit the generalizability of our findings. Furthermore, given the number of predictors included in our model, there is an inherent risk of overfitting, which can result in unstable estimates and reduced applicability of the results to other populations. We are currently enrolling a new series of patients, with continuous EEG being performed in the acute phase of TBI. Several issues complicated the multi-visit prospective study of TBI patients. Patients often do not feel the need to return for visits, especially if they have no new complaints. The challenge is ongoing, with difficulties increasing proportionally to the number of recruited patients. Another factor is that brain injury patients need a family member to attend medical visits, particularly to report potential epileptic seizures, ideally someone who witnessed the seizure. Despite these challenges and visit delays of 2–3 months, the study was completed, providing important data on the epidemiology and incidence of PTE in a developing country.

## Conclusion

PTE affects a significant proportion of TBI patients, with no efficient preventative treatments available. Developing predictive biomarkers is crucial to guide patients and families. Understanding epileptogenesis is key to prevention. Routine EEG, a low-cost, accessible, and non-invasive test, is valuable not only for detecting epileptiform elements but also for identifying abnormalities in underlying activity.

## Data Availability

The raw data supporting the conclusions of this article will be made available by the authors, without undue reservation.

## References

[1] Maas AI Stocchetti N Bullock R. Moderate and severe traumatic brain injury in adults. Lancet Neurol. (2008) 7:728–41. doi: 10.1016/S1474-4422(08)70164-9, PMID: 18635021

[2] James SL Bannick MS Montjoy-Venning WC Theadom A Ellenbogen RG Montjoy-Venning W. Global, regional, and national burden of traumatic brain injury and spinal cord injury, 1990-2016: a systematic analysis for the global burden of disease study 2016. Lancet Neurol. (2019) 18:56–87. doi: 10.1016/S1474-4422(18)30415-0, PMID: 30497965 PMC6291456

[3] Selassie AW Zaloshnja E Langlois JA Miller T Jones P Steiner C. Incidence of long-term disability following traumatic brain injury hospitalization, United States, 2003. J Head Trauma Rehabil. (2008) 23:123–31. doi: 10.1097/01.HTR.0000314531.30401.39, PMID: 18362766

[4] Gustavsson A Svensson M Jacobi F Allgulander C Alonso J Beghi E. Cost of disorders of the brain in Europe 2010. Eur Neuropsychopharmacol. (2011) 21:718–79. doi: 10.1016/j.euroneuro.2011.08.008, PMID: 21924589

[5] Majdan M Plancikova D Brazinova A Rusnak M Nieboer D Feigin V. Epidemiology of traumatic brain injuries in Europe: a cross-sectional analysis. Lancet Public Health. (2016) 1:e76–83. doi: 10.1016/S2468-2667(16)30017-2, PMID: 29253420

[6] Magalhães ALG de Barros JLVM Cardoso MG d F Rocha NP Faleiro RM de Souza LC. Traumatic brain injury in Brazil: an epidemiological study and systematic review of the literature. Arq Neuropsiquiatr. (2022) 80:410–23. doi: 10.1590/0004-282X-ANP-2021-0035 35476074 PMC9173215

[7] Englander J Bushnik T Duong TT Cifu DX Zafonte R Wright J. Analyzing risk factors for late posttraumatic seizures: A prospective, multicenter investigation. Arch Phys Med Rehabil. (2003) 84:365–73. doi: 10.1053/apmr.2003.50022, PMID: 12638104

[8] Slobounov S Sebastianelli W Hallett M. Residual brain dysfunction observed one year post-mild traumatic brain injury: combined EEG and balance study. Clin Neurophysiol. (2012) 123:1755–61. doi: 10.1016/j.clinph.2011.12.022, PMID: 22361265 PMC3513284

[9] Beghi E Carpio A Forsgren L Hesdorffer DC Malmgren K Sander JW. Recommendation for a definition of acute symptomatic seizure. Epilepsia. (2010) 51:671–5. doi: 10.1111/j.1528-1167.2009.02285.x, PMID: 19732133

[10] Sødal HF Storvig G Tverdal C Robinson HS Helseth E Taubøll E. Early post-traumatic seizures in hospitalized patients with traumatic brain injury. Acta Neurol Scand. (2022) 146:485–91. doi: 10.1111/ane.13670, PMID: 35833266 PMC9796016

[11] Fisher RS Acevedo C Arzimanoglou A Bogacz A Cross JH Elger CE. ILAE official report: a practical clinical definition of epilepsy. Epilepsia. (2014) 55:475–82. doi: 10.1111/epi.12550, PMID: 24730690

[12] Salazar AM Grafman J. Post-traumatic epilepsy: clinical clues to pathogenesis and paths to prevention. Handb Clin Neurol. (2015) 128:525–538. doi: 10.1016/B978-0-444-63521-1.00033-9 25701905

[13] Fordington S Manford M. A review of seizures and epilepsy following traumatic brain injury. J Neurol. (2020) 267:3105–11. doi: 10.1007/s00415-020-09926-w, PMID: 32444981 PMC7501105

[14] Siig Hausted H Nielsen JF Odgaard L. Epilepsy after severe traumatic brain injury: frequency and injury severity. Brain Inj. (2020) 34:889–94. doi: 10.1080/02699052.2020.1763467, PMID: 32506958

[15] Perucca P Smith G Santana-Gomez C Bragin A Staba R. Electrophysiological biomarkers of epileptogenicity after traumatic brain injury. Neurobiol Dis. (2019) 123:69–74. doi: 10.1016/j.nbd.2018.06.002, PMID: 29883622 PMC6281777

[16] Chen H Koubeissi MZ. Electroencephalography in epilepsy evaluation. Continuum. (2019) 25:431–53. doi: 10.1212/CON.0000000000000705, PMID: 30921017

[17] Lewine JD Plis S Ulloa A Williams C Spitz M Foley J. Quantitative EEG biomarkers for mild traumatic brain injury. J Clin Neurophysiol. (2019) 36:298–305. doi: 10.1097/WNP.0000000000000588, PMID: 31094883

[18] Kim JA Boyle EJ Wu AC Cole AJ Staley KJ Zafar S. Epileptiform activity in traumatic brain injury predicts post-traumatic epilepsy. Ann Neurol. (2018) 83:858–62. doi: 10.1002/ana.25211, PMID: 29537656 PMC5912971

[19] Jennett B van de Sande J. EEG prediction of post-traumatic epilepsy. Epilepsia. (1975) 16:251–6. doi: 10.1111/j.1528-1157.1975.tb06055.x, PMID: 807472

[20] Chen Y Li S Ge W Jing J Chen HY Doherty D. Quantitative epileptiform burden and electroencephalography background features predict post-traumatic epilepsy. J Neurol Neurosurg Psychiatry. (2023) 94:245–9. doi: 10.1136/jnnp-2022-329542, PMID: 36241423 PMC9931627

[21] Fisher RS Cross JH D’Souza C French JA Haut SR Higurashi N. Instruction manual for the ILAE 2017 operational classification of seizure types. Epilepsia. (2017) 58:531–42. doi: 10.1111/epi.13671 28276064

[22] Hirsch LJ Fong MWK Leitinger M LaRoche SM Beniczky S Abend NS. American clinical neurophysiology society’s standardized critical care EEG terminology: 2021 version. J Clin Neurophysiol. (2021) 38:1–29. doi: 10.1097/WNP.0000000000000806, PMID: 33475321 PMC8135051

[23] Lowenstein DH. Epilepsy after head injury: an overview. Epilepsia. (2009) 50:4–9. doi: 10.1111/j.1528-1167.2008.02004.x, PMID: 19187288

[24] Annegers JF Hauser WA Coan SP Rocca WA. A population-based study of seizures after traumatic brain injuries. N Engl J Med. (1998) 338:20–4. doi: 10.1056/NEJM199801013380104, PMID: 9414327

[25] Annegers JF Coan SP. The risks of epilepsy after traumatic brain injury. Seizure. (2000) 9:453–7. doi: 10.1053/seiz.2000.0458, PMID: 11034867

[26] Temkin NR. Risk factors for posttraumatic seizures in adults. Epilepsia. (2003) 44:18–20. doi: 10.1046/j.1528-1157.44.s10.6.x, PMID: 14511390

[27] Temkin NR Dikmen SS Wilensky AJ Keihm J Chabal S Winn HR. A randomized, double-blind study of phenytoin for the prevention of post-traumatic seizures. N Engl J Med. (1990) 323:497–502. doi: 10.1056/NEJM199008233230801, PMID: 2115976

[28] Hauser WA Annegers JF Kurland LT. Incidence of epilepsy and unprovoked seizures in Rochester, Minnesota: 1935–1984. Epilepsia. (1993) 34:453–8. doi: 10.1111/j.1528-1157.1993.tb02586.x, PMID: 8504780

[29] Verellen RM Cavazos JE. Post-traumatic epilepsy: an overview. Therapy. (2010) 7:527–31. doi: 10.2217/thy.10.57, PMID: 24761136 PMC3992621

[30] Weber C Andreassen JS Isles S Thorsen K McBride P Søreide K. Incidence, mechanisms of injury and mortality of severe traumatic brain injury: an observational population-based cohort study from New Zealand and Norway. World J Surg. (2022) 46:2850–7. doi: 10.1007/s00268-022-06721-8, PMID: 36064869 PMC9636291

[31] Golub VM Reddy DS. Post-traumatic epilepsy and comorbidities: advanced models, molecular mechanisms, biomarkers, and novel therapeutic interventions. Pharmacol Rev. (2022) 74:387–438. doi: 10.1124/pharmrev.121.000375, PMID: 35302046 PMC8973512

[32] Angeleri F Majkowski J Cacchiò G Sobieszek A D'Acunto S Gesuita R. Posttraumatic epilepsy risk factors: one-year prospective study after head injury. Epilepsia. (1999) 40:1222–30. doi: 10.1111/j.1528-1157.1999.tb00850.x, PMID: 10487184

